# Non-cirrhotic Idiopathic portal hypertension in systemic sclerosis patients: report of one case and a systematic review of previous case reports

**DOI:** 10.1186/s42358-025-00442-x

**Published:** 2025-03-04

**Authors:** Felipe Souza da Silva, João Victor de Pinho Costa, Carlos Alberto dos Santos Júnior, Érika Emmylaine dos Santos, Ailton José de Castro Júnior, Ana Cecília de Sena Oliveira, Flávia Patrícia Sena Teixeira Santos, Adriana Maria Kakehasi, Débora Cerqueira Calderaro

**Affiliations:** 1 Faculdade de Medicina da Universidade Federal de Minas, Avenida Professor Alfredo Balena, 190, Belo Horizonte, Minas Gerais 30130-100 Brazil; 2 Hospital das Clínicas da Universidade Federal de Minas Gerais, Belo Horizonte, Brazil

**Keywords:** Systemic sclerosis, Idiopathic portal hypertension, Doppler ultrasound, Transient elastography, Liver biopsy

## Abstract

**Background:**

The overlap of non-cirrhotic idiopathic portal hypertension (NCIPH) and systemic sclerosis (SSc) is rare. This article reports one case of a patient with SSc developing NCIPH and presents a systematic review of previously reported cases.

**Methods:**

CARE guidelines and the PRISMA statement were applied.

**Results:**

We report the case of a 52 year-old woman, presenting, in 2015, diffuse cutaneous scleroderma (SSc), treated with oral prednisolone and monthly intravenous cyclophosphamide. Three months later, she developed a scleroderma renal crisis, requiring hemodialysis for 18 months. Since 2017 she has not been on immunosuppressive treatment for SSc, the cutaneous involvement improved, and she has a stable Kdigo 3 chronic kidney disease. In 2019, she developed ascites. During investigation, NCIPH leading to small and medium esophageal varices and collateral circulation was diagnosed. Currently, the patient is undergoing prophylactic endoscopic band ligation of the esophageal varices and presents a stable condition. In the systematic review, 18 papers reporting 20 cases of NCIPH associated with SSc were included. Seventeen (81%) patients were women, with [Mean (SD)]: 56.71 (12.97) years. Classification of SSc was (*N* = 15): 10 limited, 4 diffuse, and 1 sin scleroderma. Clinical presentation of NCIPH was esophageal and/or gastric varices [19 (90,5%)], ascites [10 (47,6%)], and upper gastrointestinal bleeding [9 (42,8%)]. NCIPH was treated with diuretics [*n* = 9 (42,8%)], endoscopic esophageal varices sclerosis or band ligation [*n* = 7 (35%)], and beta-blockers [*n* = 4 (19%)]. Recovery of symptoms, or stabilization of clinical condition was reported in nine patients. Despite the death of seven patients, only one was attributed to the hepatic condition.

**Conclusions:**

NCIPH has been rarely reported in SSc patients. NCIPH prognosis in SSc is good. Due to the scarcity of cases reporting the occurrence of both diseases, the characteristics of SSc patients at risk of developing NCIPH remain unclear.

## Background

Non-cirrhotic idiopathic portal hypertension (NCIPH) is a rare disorder characterized by clinical portal hypertension in the absence of a recognizable cause such as cirrhosis, schistosomiasis, or portal vein thrombosis [1]. A formal diagnosis of NCIPH is based on the following criteria: (1) presence of unequivocal signs of portal hypertension, (2) absence of cirrhosis, advanced fibrosis, or other causes of chronic liver diseases, and (3) absence of thrombosis of the hepatic veins or of the portal vein at imaging [2].

Laboratory tests often reveal a preserved or nearly normal liver function with anemia, leukopenia, and thrombocytopenia due to splenomegaly with hypersplenism. Imaging studies reveal signs of portal hypertension. The most frequent clinical presentation is variceal bleeding. Liver biopsy is considered mandatory to rule out other causes of portal hypertension. Liver histology may only show subtle or mild changes.

Historically, several terms have been used to refer to the histologic changes in NCIPH, including hepatoportal sclerosis, noncirrhotic portal fibrosis, nodular regenerative hyperplasia (NRH), obliterative portal venopathy (OPV), and porto-sinusoidal vascular disease [3].

Survival is mainly limited by concomitant disorders. Although the pathophysiology of this entity remains unknown, it is frequently associated with underlying immunological disorders, bacterial infections, trace metal poisoning, medications, liver circulatory disturbances, and thrombotic events [4].

Systemic sclerosis (SSc) is a multisystemic autoimmune disease that mainly affects the connective tissue, characterized by the formation of tissue fibrosis, especially of the skin, the lungs and the gastrointestinal tract, in addition to diffuse vasculopathy [5]. The association between systemic sclerosis and NCIPH has been suggested, with a few cases previously reported [6].

This article aims to present one case report of a patient diagnosed with systemic sclerosis (SSc) who developed idiopathic portal hypertension (IPH), and a systematic review of the Literature including the reported cases suggesting an association between SSc and IPH.

## Methods

### Case report

Case was reported according to the CARE guidelines [7].

### Ethical permit

The patient gave informed consent according to the institutional review board.

### Systematic review

#### Data source and searches

The systematic review followed the PRISMA statement [8].

The protocol for this systematic review was hosted in PROSPERO database (PROSPERO Registration: CRD42023459217).

Literature for this systematic review has been searched electronically from September 2023 to October 2023 in PubMed, SciELO and LILACS/Virtual Health Library (VHL). Search was done using highly sensitive strategies, described as: (((“Scleroderma, Systemic“[Mesh]) OR (Sclerosis, Systemic) OR (Systemic Scleroderma) OR (Systemic Sclerosis) OR “Scleroderma, Diffuse“[Mesh]) OR (Diffuse Scleroderma) OR (Scleroderma, Progressive) OR (Progressive Scleroderma) OR (Sclerosis, Progressive Systemic) OR (Progressive Systemic Sclerosis) OR (Systemic Sclerosis, Progressive) OR (Sudden Onset Scleroderma) OR (Sclerodermas, Sudden Onset) OR (Scleroderma, Sudden Onset) OR (Sudden Onset Sclerodermas) OR (Diffuse Systemic Sclerosis) OR (Diffuse Systemic Scleroses) OR (Scleroses, Diffuse Systemic) OR (Sclerosis, Diffuse Systemic) OR (Systemic Scleroses, Diffuse) OR (Systemic Sclerosis, Diffuse) OR (Diffuse Cutaneous Systemic Sclerosis) OR “Scleroderma, Limited“[Mesh]) OR (Limited Scleroderma) OR (Limited Systemic Scleroderma) OR (Limited Systemic Sclerodermas) OR (Scleroderma, Limited Systemic) OR (Sclerodermas, Limited Systemic) OR (Systemic Scleroderma, Limited) OR (Systemic Sclerodermas, Limited) OR “CREST Syndrome“[Mesh]) OR (CREST Syndromes) OR (Syndrome, CREST) OR (Calcinosis, Raynaud’s phenomenon, Esophageal dismobility, Sclerodactyly, Telangiectasia Syndrome) OR (CRST Syndrome) OR (CRST Syndromes) OR (Syndrome, CRST) OR (Calcinosis-Raynaud Phenomenon-Sclerodactyly-Telangiectasia) OR (Calcinosis Raynaud Phenomenon Sclerodactyly Telangiectasia) OR (Phenomenon-Sclerodactyly-Telangiectasia, Calcinosis-Raynaud))) AND (((“Hypertension, Portal“[Mesh]) OR (Banti’s Disease) OR (Banti Disease) OR (Disease, Banti’s) OR (Idiopathic Portal Hypertension) OR (Hypertension, Idiopathic Portal) OR (Idiopathic Portal Hypertensions) OR (Portal Hypertension, Idiopathic) OR (Idiopathic Congestive Splenomegaly) OR (Congestive Splenomegaly, Idiopathic) OR (Idiopathic Congestive Splenomegalies) OR (Splenomegaly, Idiopathic Congestive) OR (Noncirrhotic Portal Fibrosis) OR (Fibrosis, Noncirrhotic Portal) OR (Noncirrhotic Portal Fibroses) OR (Portal Fibrosis, Noncirrhotic) OR (Idiopathic Non-Cirrhotic Portal Hypertension) OR (Idiopathic Non Cirrhotic Portal Hypertension) OR (Banti’s Syndrome) OR (Banti Syndrome) OR (Syndrome, Banti’s) OR (INCPH) OR (Porto-Sinusoidal Vascular Diseases) OR (Disease, Porto-Sinusoidal Vascular) OR (Diseases, Porto-Sinusoidal Vascular) OR (Porto-Sinusoidal Vascular Disease) OR (Porto Sinusoidal Vascular Diseases) OR (Vascular Disease, Porto-Sinusoidal))) AND (((“Case Reports“[Mesh]) OR (Case Histories) OR (Case Study) OR (Case Studies))) and described in SciELO, BVS and LILACS/Virtual Health Library (VHL) as: Scleroderma, Systemic OR Scleroderma, Diffuse OR Scleroderma, Limited OR CREST Syndrome AND Hypertension, Portal AND Case Reports.

Case Reports in English, Spanish or Portuguese were included. Additional studies were manually searched by analyzing the references from relevant articles.

#### Study selection and data extraction

Three reviewers (FSS, JVPC, CASJ) independently evaluated and scrutinized the full text articles for eligibility as per criteria for eligibility. Conflicts between reviewers were resolved by mutual consent or by involving another reviewer (DCC). Case reports of SSc patients with IPH were included. Studies reporting SSc patients with primary biliary cholangitis, hepatic cirrhosis, hepatitis or any identifiable cause of portal hypertension were excluded. The lack of information of the surveillance of other causes of portal hypertension was also an exclusion criteria.

Data extracted from the included case reports were: the year of publication, the country of residence of the patients, age, sex, ethnicity, comorbidities, clinical presentation subtype of SSc, laboratory findings, radiology, endoscopy, liver biopsy, treatment and outcomes.

#### Integration of results

The data were extracted and qualitatively synthesized.

The scarcity of cases reported, and the heterogeneity in their clinical description, investigations procedures, interventions, and outcome measures precluded the authors from performing a quantitative analysis.

### Evaluation of the risk of biases

The JBI’s critical appraisal tool checklist for case reports [9] was used for the evaluation of risk of bias in the studies included in the systematic review. Quality assessment was done by two independent investigators (FSS and CASTJ). Conflicts were resolved by mutual consent.

Final scores for the 8 appraisal criteria were obtained by calculating the arithmetic mean between the scores from both investigators.

Studies reporting 5 of the 8 appraisal criteria were considered to present an acceptable quality and were included in the review.

## Results

### Case report

In January, 2015, a 46 year-old woman was diagnosed with diffuse SSc. The disease was characterized by Raynaud phenomenon (RP), puffy fingers, severe diffuse cutaneous thickening, incipient pulmonary fibrosis (septal thickening and peripheral ground-glass opacities) with mild reduction of forced vital capacity (FVC% predicted: 78%) and DLCO (68%) in pulmonary function test (PFT), minimal pleural and pericardial effusion, no pulmonary hypertension, inflammatory myositis (proximal muscle weakness with elevated creatine phosphokinase), and esophagopathy. RP, puffy fingers, cutaneous thickening, gastroesophageal reflux and inflammatory myositis symptoms developed in December, 2014. Laboratory results revealed elevated erythrocyte sedimentation rate and C-reactive protein, and positive fine speckled nuclear antinuclear antibodies (ANA) 1:640, with negative anticentromere, anti-topoisomerase, anti-PM/Scl, anti-Ro, anti-La, anti-citrullinated peptide antibodies and rheumatoid factor.

Treatment with prednisone 60 mg/day and monthly intravenous cyclophosphamide (1 g/month) was started.

In April, 2015, the patient developed scleroderma renal crisis (severe arterial hypertension, acute kidney injury, kidney biopsy with myxoid intimal changes and fibrointimal sclerosis), treated with high doses of captopril and hemodialysis for 18 months.

During follow-up, the patient completed 6 months of intravenous cyclophosphamide, prednisone was tapered and suspended. Low-dose azathioprine was used as maintenance therapy from 2015 to 2017. Since 2017, the patient is in drug free remission. Currently, she presents stable SSc, with modified Rodnan skin score of 2 (sclerodactyly), stable interstitial lung disease (septal thickening and peripheral ground-glass opacities), normal FVC %predicted (86%), mild DLCO %predicted reduction (68%), no new digital ulcerations, tendon friction rub, arthritis, or proximal muscle weakness, and normal CKemia, erythrocyte sedimentation rate and c-reactive protein.

A chronic kidney disease Kdigo 3 established and is currently managed by a nephrologist, with kidney function follow-up and the use of anti-hypertensives including captopril.

Her current treatment consists of a proton pump inhibitor, carvedilol, antihypertensives and diuretics.

In December, 2019, she was admitted to an Internal Medicine Unit due to ascites, confirmed by computed tomography (CT). Liver function tests were in range, as well as coagulation proteins, transaminases, and cholestasis indices. HBV, HCV and HIV infections, as well as alcohol or drug abuses, were excluded. Wilson’s disease and autoimmune hepatitis surveillance were negative. Esophagogastroduodenoscopy evidenced esophageal varices with collateral circulation. Magnetic resonance cholangiography and hepatic elastography were normal. Abdominal doppler ultrasound depicted increased portal vein diameter, with portosystemic shunt and splenomegaly. After improvement of ascites with the use of diuretics, a liver needle biopsy was performed. The liver biopsy showed normal architecture, with a mild inflammatory infiltrate, and no necrosis or fibrosis. The final diagnosis was NCIPH.

The patient presents rare episodes of recurrent ascites treated with diuretics and periodically undergoes endoscopic prophylactic band ligation therapy of the esophageal varices. She is in a stable condition.

### Systematic review

**Fig. 1:**
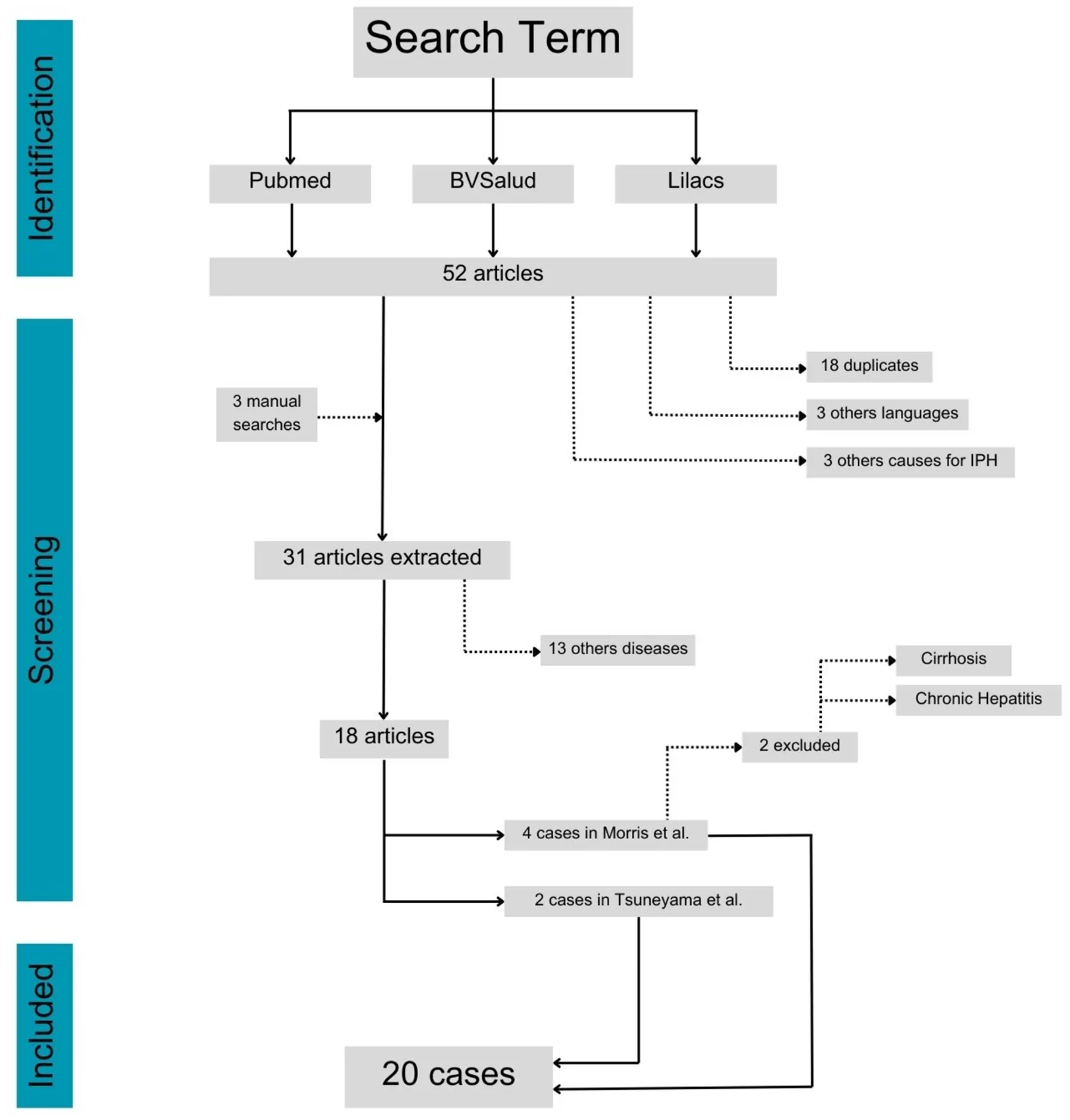
Selection of studies algorithm

Literature search retrieved 52 articles, and manual search retrieved three more papers. After exclusion of duplicates and articles not meeting inclusion criteria, or presenting exclusion criteria, 18 articles were screened and finally included for analysis in this systematic review (Fig. 1).

### Evaluating the risk of bias

The JB risk of bias for each included article is presented in Table 1. Fourteen (70%) reported cases included demographic information on age, gender and race [1, 6, 11–16, 18–24], 19 (95%) described patient’s history/timelines [1, 6, 10–24]. The patient presentation on IPH was clearly reported by 18 (90%) case reports [1, 6, 10, 11, 13–24]. The assessment methods and diagnostic tests performed, and their results were adequately depicted in all cases [1, 5, 6, 10–24], while the interventions or treatment procedures were clearly described in 19 (95%) case reports [1, 5, 6, 10–14, 16–24]. In 17 (85%) reports [1, 5, 10, 12–18, 20–24] the post-intervention clinical condition of the patients were described clearly, while only 6 (30%) [1, 16, 17, 19, 21, 22] described the occurrence of adverse events after interventions. Relevant take away lessons are reported in 16 (80%) [1, 5, 6, 11–16, 18, 20–24] cases.

**Table 1:** JBI risk of bias assessment

Cases	Criterias								Overall Appraisal
	Studies included information on age, gender and race	Studies described patient’s history or timelines	The patient presentation on IPH was clearly reported	The assessment methods and diagnostic tests performed, and their results were adequately depicted	Interventions or treatment procedures were clearly described	Describe clearly the post-intervention clinical condition of the patients	Report adverse events after interventions	Relevant take away lessons are reported	
Morris et al. [10] - Case 1	N	Y	Y	Y	Y	Y	UN	N	Included
Morris et al. [10] - Case 2	N	Y	Y	Y	Y	Y	UN	N	Included
Umeyama et al. [11]	Y	Y	Y	Y	Y	N	UN	Y	Included
Kabukari et al. [12]	N	Y	N	Y	Y	Y	UN	Y	Included
Manuel et al. [13]	N	Y	Y	Y	Y	Y	UN	Y	Included
Garcia et al. [14]	Y	Y	Y	Y	Y	Y	UN	Y	Included
Tsuneyama et al. [15] - Case 1	Y	Y	Y	Y	N	Y	UN	Y	Included
Tsuneyama et al. [15] - Case 2	Y	Y	Y	Y	Y	Y	UN	Y	Included
Kogawa et al. [16]	Y	Y	Y	Y	Y	Y	Y	Y	Included
Moschos et al. [17]	N	Y	Y	Y	Y	Y	Y	UN	Included
Takagi et al. [18]	Y	Y	Y	Y	Y	Y	UN	Y	Included
Samanta et al. [1]	Y	Y	Y	Y	Y	Y	Y	Y	Included
Espinosa et al. [19]	Y	Y	Y	Y	Y	UN	Y	UN	Included
Kamel et al. [20]	Y	Y	Y	Y	Y	Y	UN	Y	Included
Abrams et al. [21]	Y	Y	Y	Y	Y	Y	Y	Y	Included
Gao et al. [3]	N	Y	Y	Y	Y	N	N	Y	Included
Saigusa et al. [22]	Y	Y	Y	Y	Y	Y	Y	Y	Included
Yamamoto et al. [23]	Y	Y	Y	Y	Y	Y	UN	Y	Included
Colaci et al. [2]	Y	N	N	Y	Y	Y	UN	Y	Included
Hitawala et al. [24]	Y	Y	Y	Y	Y	Y	UN	Y	Included
	14 (70%)	19 (95%)	18 (90%)	20 (100%)	19 (95%)	17 (85%)	6 (30%)	16 (80%)	

Y, Yes; N, No; UN, Undefined

### Evidence synthesis

According to the inclusion criteria of the systematic review, only cases reporting that other causes of portal hypertension (e.g. viral or autoimmune hepatitis, primary biliary cirrhosis, HIV infection, thrombophilias) were excluded, according to laboratory or hepatic biopsy work-up, were selected for this review.

The description of the characteristics of the case reports are shown in Table 2. The 20 cases previously reported, and the case presented in this study, were included in the evidence synthesis.

**Table 2:** Summary of reported cases

	Sex	Age	Extension of cutaneous thickening	SSc manifestations	SSc serology	NCIPH manifestations	NCIPH exams	Management of NCIPH	NCIPH outcomes
Morris et al. [10] - Case 1	Female	53	nd	Raynaud’s phenomenon, dysphagia, calcinosis cutis, telangiectasias	ANA	Esophageal varices, upper gastrointestinal bleeding	Barium swallow, splenoportography, liver biopsy	End-to-side porto-caval anastomosis	Abscence of complications in 12 months
Morris et al. [10] - Case 2	Female	49	nd	Raynaud’s phenomenon, sclerodactily	ANA	Anemia, ascites esophageal varices, upper gastrointestinal bleeding	Barium swallow, splenoportography, liver biopsy	End-to-side porto-caval anastomosis, diuretics	Recovered from ascites
Umeyama et al. [11]	Female	41	Limited Cutaneous Scleroderma	Microstomy, sclerodactily, esophagopathy	ANA	Pancytopenia, hepatosplenomegaly, upper gastrointestinal bleeding, collateral circulation, esophagogastric varices	Upper gastrointestinal endoscopy, splenoportography	Splenectomy, esophageal transection	nd
Kabukari et al. [12]	Female	33	nd	Raynaud’s phenomena, sclerodactily, digital pitting scars, microstomy, interstitial lung disease, chronic kidney disease	ANA, anti-U1-RNP, Anti-SSA-Ro, Anti-SSB-la	Thrombocytopenia, hepatoesplenomegaly, ascites, collateral circulation	Percutaneous transhepatic portography with venous pressure measurement, liver biopsy	Prednisolone 30 to 60 mg, intravenous immunoglobulin, diuretics	Death by renal and hepatic disfunction
Manuel et al. [13]	Female	54	Limited Cutaneous Scleroderma	Calcinosis cutis, raynaud’s phenomenon, esophagopathy, sclerodactily, telangiectasias	ANA, anticentromere	Anemia, thrombocytopenia, esophageal varices, upper gastrointestinal bleeding, splenomegaly, portal hypertension gastropathy	Abdominal doppler ultrasound, upper gastrointestinal endoscopy, arteriography, liver biopsy	Endoscopic sclerosis of esophageal varices, propranolol, portosystemic shunt	Without gastrointestinal bleeding in 1 year
Garcia et al. [14]	Male	59	Diffuse Cutaneous Scleroderma	nd	None	Hepatomegaly, splenomegaly, telangiectasias, palmar erythema, esophageal varices, portal hypertension gastropathy, thrombocytopenia	Abdominal ultrasound, computed tomography, resonance, upper gastrointestinal endoscopy, liver biopsy, portography	nd	nd
Tsuneyama et al. [15] - Case 1	Female	61	Diffuse Cutaneous Scleroderma	nd	ANA	Anemia, ascites, splenomegaly esophageal varices, upper gastrointestinal bleeding	Liver biopsy	nd	Death by pneumonia
Tsuneyama et al. [15] - Case 2	Female	58	Diffuse Cutaneous Scleroderma	Interstitial lung disease, telangiectasias	ANA, anti-SCL70	Anemia, esophageal varices, splenomegaly, ascites	Liver biopsy	Splenectomy, esophageal dissection	Death by sepsis 4 months later
Kogawa et al. [16]	Female	72	Limited Cutaneous Scleroderma	Raynaud’s phenomenon, interstitial lung disease, telangiectasias	ANA, anti-SCL70, anticentromere, anti-SSA-Ro	Esophageal varices, anemia, thrombocytopenia	Computed tomography, liver biopsy, upper gastrointestinal endoscopy	Oral corticosteroids	Death by respiratory failure due to de ILD exacerbation
Moschos et al. [17]	Male	82	nd	Interstitial lung disease	nd	Mild anemia, esophageal varices, upper gastrointestinal bleeding	Upper gastrointestinal endoscopy, ultrassound, computed tomography, Liver biopsy	Propranolol, azathioprine, spironolactone, prednisolone, lansoprazole, endoscopic varices sclerotherapy and banding, Sengstaken blakemore tube, intravenous glypressin	Stable in 2 months
Takagi et al. [18]	Female	62	nd	Raynaud’s phenomenon, sclerodactily, digital pitting scars, interstitial lung disease	ANA, anti-U1-RNP	Pancytopenia, ascites, pleural effusion, splenomegaly esophageal varices	Ultrassound, computed tomography, magnetic resonance imaging, liver biopsy	Prednisolone 30 mg, diuretics	Stable 3 years later
Samanta et al. [1]	Male	65	Limited Cutaneous Scleroderma	Raynaud’s phenomenon, calcinosis cutis, sclerodactily, esophageal disease, telangiectasias	ANA, anticentromere, Rheumatoid factor	Esophageal varices, upper gastrointestinal bleeding, portal gastropathy	Ultrassound, contrasted computed, tomography, liver biopsy, upper gastrointestinal endoscopy	Hemodynamic resuscitation, endoscopic esophageal varices band ligation, intravenous glypressin	nd
Espinosa et al. [19]	Female	38	Limited Cutaneous Scleroderma	Raynaud’s phenomenon, sclerodactily, interstitial lung disease, telangiectasias, arthritis, esophagopathy, pulmonary hypertension, peripheral neuropathy	ANA, anticentromere	Esophageal varices, splenomegaly	Ultrassound, liver biopsy, magnetic resonance imaging	Endoscopic ligation of esophageal varices, prophylactic treatment with 40 mg of propranolol every 12 h.	nd
Kamel et al. [20]	Female	57	Sin scleroderma	Pulmonary arterial, hypertension	ANA, anti-ds-DNA, anticentromere, Anti-polimerase III	Gastroesophageal varices	Computed tomography, liver biopsy	Diuretics, ambrisentan, sildenafil, furosemide, oxygen supplementation, hydroxychloroquine	Cardiac sudden death one year later
Abrams et al. [21]	Male	59	Limited Cutaneous Scleroderma	CREST syndrome (calcinosis, Raynaud’s phenomenon, esophagopathy, sclerodactily, telangiectasias)	ANA	Ascites, esophageal varices, pleural effusions	Abdominal ultrassound, liver biopsy	Diuretics, thoracocentesis, paracentesis, transjugular intrahepatic portal systemic shunt study (TIPS)	Improvement of ascitis and pleural effusion after TIPS. Encephalophaty controlled with medication
Gao et al. [3]	Female	51	nd	Raynaud’s phenomenon, sclerodactily, puffy fingers	ANA, anticentromere	Anemia, esophageal varices, upper gastrointestinal bleeding	Doppler ultrasound, computed tomography	Splenectomy	nd
Saigusa et al. [22]	Female	73	Limited Cutaneous Scleroderma	Raynaud’s phenomenon, sclerodactily, digital ulcers, lower limbs ulcers, pulmonary arterial hypertension	ANA, anticentromere, anti-SSA-Ro	Anemia, esophageal varices	Upper gastrointestinal endoscopy, computed tomography	Endoscopic esophageal varices band ligation	Death by deterioration of her condition
Yamamoto et al. [23]	Female	76	Limited Cutaneous Scleroderma	Raynaud’s phenomenon, sclerodactily	ANA, anticentromere, Anti-ds-DNA	Mild anemia, thrombocytopenia, ascites, esophageal varices	Computed tomography, upper gastrointestinal endoscopy, magnetic resonance imaging, liver biopsy	Diuretics	No symptoms of ascitis or SSc
Colaci et al. [2]	Female	61	Limited Cutaneous Scleroderma	Telangiectasias, esophagopathy	ANA, anticentromere	Ascites collateral circulation	Liver biopsy, computed tomography, upper gastrointestinal endoscopy	Diuretics	Stable for 1 year follow-up. Death by sepsis later
Hitawala et al. [24]	Female	37	Limited Cutaneous Scleroderma	Raynaud’s phenomenon, sclerodactily, telangiectasias, interstitial lung disease	ANA, anticentromere, Rheumatoid factor	Splenomegaly, pancytopenia, gastroesophageal varices, upper gastrointestinal bleeding, collateral circulation, ascites	Ultrassound, computed tomography, upper gastrointestinal endoscopy, hepatic elastography, magnetic resonance imaging, liver biopsy	Endoscopic esophageal varices band ligation, carvedilol	Stable 1 year later
Present Case	Female	50	Diffuse Cutaneous Scleroderma	Raynaud’s phenomenon, puffy fingers, myositis, interstitial lung disease, esophageal reflux, scleroderma renal crisis	ANA	Mild anemia, ascites, esophageal varices, collateral circulation	Ultrassound, computed tomography, magnetic resonance imaging, upper gastrointestinal endoscopy, hepatic elastography, liver biopsy	Antihypertensives diuretics, endoscopic esophageal varices band ligation	Stable 5 years later

ANA, antinuclear antibody; nd, not declared; Ssc, Systemic sclerosis; TIPS, transjugular intrahepatic porto-systemic shunt

The reported cases included in this study were from Brazil (*n* = 2, one currently reported and one from the systematic review) [19], China (*n* = 1) [3], Italy (*n* = 1) [24], Japan (*n* = 8) [11, 12, 15, 16, 18, 22, 23], Spain (*n* = 2) [13, 14], United Kingdom (*n* = 4) [1, 10, 17] and the United States of America (*n* = 3) [20, 21, 24].

Seventeen (81%) patients were women, with [Mean (SD)]: 56.71 (12.97) years of age. Classification of SSc was described in only 15 cases: 10 had limited SSc, 4 presented diffuse SSc, and 1 was classified with sin scleroderma SSc.

Besides cutaneous thickening, the SSc manifestation reported were Raynaud’s phenomenon [14 (66,7%)], sclerodactyly [12 (57,1%)], telangiectasias [9 (42,8%)], esophageal disease [8 (38,1%)], cutaneous calcinosis [4 (19%)], digital ulcers and pitting scars [3 (14,3%)], microstomy [2 (9,5%)], puffy fingers [2 (9,5%)], lower limbs ulcers [1 (4,7%)], interstitial lung disease [8 (38,1%)], pulmonary hypertension [3 (14,2%)], peripheral neuropathy [1 (4,7%)], scleroderma renal crisis [1 (4,7%)], chronic kidney disease [1 (4,7%)], arthritis [1 (4,7%)], and myositis [1 (4,7%)].

NCIPH diagnosis occurred after or concurrently with SSc diagnosis in all cases. Clinical presentation of NCIPH included ascites [10 (47,6%)], esophageal and/or gastric varices [19 (90,5%)], upper gastrointestinal bleeding [9 (42,8%)], portal gastropathy [3 (14,3%)], collateral circulation [5 (23,8%)], splenomegaly [8 (38,1%)], palmar erythema [1 (4,7%)], and pleural effusion [1 (4,7%)].

Hematologic findings were anemia in 10 (47,6%), thrombocytopenia in 5 (23,8%) or pancytopenia in 3 (14,2%) cases. Transaminases were in normal range or only mildly elevated in all cases.

Imaging diagnostic work-up for IPH included liver biopsy [*n* = 18 (85,7%)], CT [*n* = 12 (57,1%)], upper gastrointestinal endoscopy [*n* = 11 (52,4%)], abdominal ultrasound [*n* = 10 (47,6%)], magnetic resonance imaging [*n* = 6 (28,6%)], portography [*n* = 5 (23,8%)], hepatic elastography [*n* = 2 (9,5%)], barium swallow [*n* = 2 (9,5%)], and arteriography [*n* = 1 (4,7%)].

IPH was treated with diuretics [*n* = 9 (42,8%)], beta-blockers [*n* = 4 (19%)], endoscopic esophageal varices sclerosis or band ligation [*n* = 7 (35%)], portocaval anastomosis [*n* = 6 (30%)], Transjugular intrahepatic porto-systemic shunt (TIPS) [*n* = 1 (5%)], splenectomy [3 (14,3%)]. Treatment with corticosteroids and/or immunosuppressants/immunomodulators was reported respectively in 5 and 2 cases (azathioprine 1, hydroxychloroquine 1).

Reported outcomes were not disclosed in four cases. Recovery of symptoms attributable to IPH, or stabilization of clinical condition were reported in nine patients. Seven (33%) patients died, mainly by infectious complications/sepsis (*n* = 3). Only one death was attributed to the hepatic disease.

## Discussion

This study describes the rare occurrence of NCIPH in SSc patients, since it reports one new case and the systematic review of 20 previously reported cases. Despite the scarcity of this presentation, these case reports highlight the presentation of other hepatic disorders in SSc patients, despite primary biliary cirrhosis, primary sclerosing cholangitis and autoimmune hepatitis. Thus, the possible association between SSc and hepatic disorders dictates the inclusion of a liver evaluation in the periodic work-up of SSc patients, by means of hepatic liver enzymes and ultrasound imaging of the abdomen.

NCIPH refers to a heterogeneous group of liver disorders that primarily affect the liver vascular system and that are classified anatomically on the basis of site of resistance to blood flow, as pre-hepatic, hepatic (pre-sinusoidal, sinusoidal or post-sinusoidal) and post-hepatic. Schistosomiasis, biliary diseases, viral hepatitis, alcoholic liver damage, Budd-Chiari syndrome and other numerous diseases have been cited as causes. Autoimmune diseases, as rheumatoid arthritis, systemic lupus erythematosus and systemic sclerosis have also been associated with NCIPH [25].

The more frequent clinical presentation of NCIPH is gastroesophageal varices, with upper gastrointestinal bleeding or not, collateral circulation, splenomegaly, ascites and, rarely, hepatic encephalopathy, which is in line with the symptoms reported in the cases of SSc patients with NCIPH. The outcomes of NCIPH are mostly determined by age and the course of the underlying disease. The main complication of NCIPH is the gastrointestinal bleeding from the rupture of esophagogastric varices. In the absence of large prospective and comparative studies in patients with NCIPH, current guidelines on vascular liver diseases suggest managing this complication according to the guidelines of cirrhotic portal hypertension. Non-specific beta-adrenergic blockade or endoscopic band ligation are used for primary prophylaxis and their combination for secondary prophylaxis in patients with NCIPH. TIPS is to consider a valid option for the patients with variceal bleeding not controlled by medical and endoscopic treatment. Mortality due to variceal bleeding is significantly lower in NCIPH patients than that observed in cirrhotic patients, likely because of a preserved liver function. Ascites is not a frequent complication of NCIPH, and it usually occurs during decompensating events such as infections or variceal bleeding. The management of ascites is the same of cirrhotic patients. Hepatic encephalopathy, both overt and minimal, is also a complication of NCIPH, much less frequent than in cirrhosis [25].

A possible link between SSc is the diffuse vasculopathy with endothelial dysfunction leading to the development of tissue fibrosis and NCIPH. It was assumed that, after a hypothetical trigger to the intrahepatic microcirculation, the obliterative portal damage due to hepatoportal sclerosis might lead to the increase of vascular resistance, and to portal hypertension [4]. Moreover, anti-endothelial antibodies (AECA), expressed in some patients with NCIPH damage endothelial cells of portal vessels, producing dense deposits of elastic fibers around the peripheral ramifications of the portal vein, leading to portal hypertension [26]. AECA were also found and directly correlated with vascular injury and endothelial damage in SSc patients, by means of antibody-dependent cellular apoptosis that stimulated the microvasculature to release pro-inflammatory and pro-fibrotic cytokines [27, 28]. Schouten et al. have proposed the occurrence of repeated microthrombosis in the small or medium portal vein branches, and the enhanced fibrogenesis occurring in SSc, as plausible triggers for NCIPH [29]. Other possibility is that abnormal immune responses and vascular damage that appear to be central to the pathogenesis of SSc can sometimes involve small portal veins within the liver causing NCIPH [1]. NCIPH has also been reported in patients with inflammatory bowel disease taking azathioprine in long-term use, with reported incidence rates of 0.5% and 1.25% over 5 years and 10 years, respectively [30]. In the case reported, patient used azathioprine in low doses and for only two years, thus, authors believe NCIPH is more probably associated with SSc than this medication.

Potential triggers leading to IPH in SSc patients, that should be excluded before diagnosing NCIPH, are the exposure to drugs and toxics (e.g. alcohol), the presence of hepatic cirrhosis of other etiologies, infection by hepatotropic or HIV viruses, schistosomiasis, the overlap with other autoimmune hepatic diseases (e.g., autoimmune hepatitis, primary biliary cirrhosis or primary sclerosing cholangitis), and the presence of thrombophilias [25].

In the synthesized cases of SSc patients presenting NCIPH, there was a majority of women, presenting limited cutaneous SSc, from different countries, and frequent SSc systemic manifestations. Thus, as NCIPH in SSc represents a condition reported anecdotally, the characteristics of patients at risk of developing this condition may not be drawn.

The therapeutic approach to SSc patients with NCIPH remains to be defined. In our case and in most of the previously reported cases, therapy addressed only the treatment or prevention of complications of NCIPH. Only a few patients reported in these previous studies were receiving corticosteroids and/or immunosuppressants in order to control SSc-related inflammation and fibrosis, thus, their impact on NCIPH needs further investigation [12, 16–18].

This article brings one case report and the systematic review of previously reported case reports of the rare occurrence of NCIPH in SSc patients, highlighting its presentation, complications, and management. Physicians and health care workers caring for SSc patients must be aware of this rare hepatic manifestation occurring in these patients.

The main limitations of this work are the scarcity of cases reported in the literature and the heterogeneity of them, which precluded the authors from drawing any conclusions on the pathophysiology of the association between SSc and NCIPH, and the clinical profile of SSc patients at risk for developing NCIPH that might benefit from increased surveillance. The scarcity in the report or SSc characteristics and of the hepatic compromise in some cases included in the systematic review, which may associate with some uncertainty regarding SSc and NCIPH diagnosis, is also a limitation. Moreover, since some drugs such as azathioprine have been rarely associated with NCIPH, the lack of sufficient information regarding the patients’ medication histories in most case reports included in this review represents a significant limitation.

## Conclusions

NCIPH is a disorder of unknown etiology, characterized by a hepatic venous gradient higher than normal range. Systemic sclerosis is an autoimmune disease characterized by microvascular damage, generalized fibrosis in different organs and dysregulation of innate immunity. Although further studies are necessary to shed light on both SSc and NCIPH pathogenesis, evidence of shared mechanisms of vascular fibrosis may be a useful source of hypothesis to the understanding of possible shared identity and/or causal relationship between them. Due to the scarcity of cases reporting the occurrence of NCIPH in SSc patients, the characteristics of the patients at risk of developing this hepatic manifestation are still unclear. The prognosis of NCPIH in SSc apparently is good. Health care professionals caring for these patients must be aware of this rare association.

## Data Availability

No datasets were generated or analysed during the current study.

## Abbreviations

NCIPH: Non-cirrhotic idiopathic portal hypertension
NRH: Nodular regenerative hyperplasia
OPV: Obliterative portal venopathy
Ssc: Systemic sclerosis
IPH: Idiopathic portal hypertension
ANA: Antinuclear antibodies
CT: Computed tomography
TIPS: Transjugular intrahepatic porto-systemic shunt
AECA: Anti-endothelial antibodies
